# Understanding the role of myoglobin content in Iberian pigs fattened in an extensive system through analysis of the transcriptome profile

**DOI:** 10.1111/age.13195

**Published:** 2022-03-30

**Authors:** Miguel Ángel Fernández‐Barroso, Juan María García‐Casco, Yolanda Núñez, Luisa Ramírez‐Hidalgo, Gema Matos, María Muñoz

**Affiliations:** ^1^ Centro Nacional de I+D del Cerdo Ibérico INIA‐CSIC Zafra Spain; ^2^ Departamento de Mejora Genética Animal INIA‐CSIC Madrid Spain; ^3^ Sánchez Romero Carvajal—Jabugo SRC Huelva Spain

**Keywords:** Iberian pig, myoglobin content, RNA‐seq, transcriptome and functional analysis

## Abstract

Meat color is the first perceived sensory feature and one of the most important quality traits. Myoglobin is the main pigment in meat, giving meat its characteristic cherry‐red color, highly appreciated by the consumers. In the current study, we used the RNA‐seq technique to characterize the longissimus dorsi muscle transcriptome in two groups of Iberian pigs with divergent breeding values for myoglobin content. As a result, we identified 57 differentially expressed genes and transcripts (DEGs). Moreover, we have validated the RNA‐seq expression of a set of genes by quantitative PCR (qPCR). Functional analyses revealed an enrichment of DEGs in biological processes related to oxidation (*HBA1*), lipid metabolism (*ECH1*, *PLA2G10*, *PLD2*), inflammation (*CHST1*, *CD209*, *PLA2G10*), and immune system (*CD209*, *MX2*, *LGALS3*, *LGALS9*). The upstream analysis showed a total of five transcriptional regulatory factors and eight master regulators that could moderate the expression of some DEGs, highlighting SPI1 and MAPK1, since they regulate the expression of DEGs involved in immune defense and inflammatory processes. Iberian pigs with high myoglobin content also showed higher expression of the *HBA1* gene and both molecules, myoglobin and hemoglobin, have been described as having a protective effect against oxidative and inflammatory processes. Therefore, the *HBA1* gene is a very promising candidate gene to harbor polymorphisms underlying myoglobin content, whereby further studies should be carried out for its potential use in an Iberian pig selection program.

## INTRODUCTION

Meat color is considered one of the most important meat quality traits and the first attribute that is sensorially perceived, influencing the purchase decision of consumers (Mancini & Hunt, 2005). A bright cherry‐red color is normally used as a healthy indicator in fresh meat, while PSE (pale, soft, exudative) or DFD (dark, firm, dry) meats do not satisfy the consumer preferences (Yu et al., 2017).

Myoglobin (MB) is the main heme sarcoplasmic protein responsible for oxygen transport and the principal pigment related to the red color of the meat (Suman & Poulson, 2013). Besides, MB is involved in the oxidative phosphorylation (Wittenberg & Wittenberg, 2003), as well as in the binding and delivery of oxygen to the mitochondria in the skeletal muscle (Suman & Poulson, 2013). Structurally, MB is a monomeric heme protein composed of a heme prosthetic group and a globin protein (Suman & Poulson, 2013). The heme group, which characterizes MB as a pigment, absorbs visible light through its double bonds and contains an iron atom that can be present in reduced (ferrous/Fe^2+^) or oxidized (ferric/Fe^3+^) form. The heme group can reversibly bind to ligands such as oxygen, carbon monoxide or nitric oxide. Therefore, there are four redox states of MB: deoxymyoglobin (reduced, DMB), oxymyoglobin (oxygenated, OMB), metmyoglobin (oxidized, MetMB), and carboxymyoglobin (COMB; Mancini & Hunt, 2005). OMB gives to the meat, a bright cherry‐red color, critical for consumer acceptance. DMB provides purplish‐red color and MetMB produces a brown color on meat. These four redox forms of MB can be identified spectrophotometrically and their absorbance spectra range between 500 and 600 nm, with 525 nm being the point at which the absorption spectral curves of the four forms converge (Tang et al., 2004).

The myoglobin content is influenced by different factors such as the species, breed, metabolic profile of the muscle (oxidative or glycolytic), age, and production system (indoor or outdoor; Olsson & Pickova, 2005; Ventanas et al., 2005; Yu et al., 2017). In fact, several authors have reported that the meat from pigs handled in open‐air extensive systems has a higher myoglobin content (Ventanas et al., 2005), being redder and paler (Pugliese et al., 2005). Moreover, from a genetic point of view, the heritability of this trait estimated both in lean and heavy pig breeds showed moderate values. Newcom et al. (2004) estimated an average heritability of 0.27 for MB in seven different breeds, while Fernández‐Barroso et al. (2020a) estimated a value of 0.15 in the Iberian breed. Kim et al. (2010) found that MB content was phenotypically correlated (*r* = 0.45) with the a* color parameter (measured by colorimeter) in crossbred pigs between Korean native black pig and Landrace, and Newcom et al. (2004) and Fernández‐Barroso et al. (2020a) estimated a positive genetic correlation between MB and a* of 0.23 and 0.94 respectively. In addition, some polymorphisms have been identified in candidate genes such as *CASP9* and *PRKAG3* (Fernández‐Barroso et al., 2020a; Lindahl et al., 2004) that affect MB content.

The Iberian pig breed is characterized by having a high‐quality meat and greatly appreciated dry‐cured products, with an elevated economic value in the market. The quality of its products is favored by its particular characteristics, such as voracious appetite, high adipogenic potential and protein turnover ratio, and low deposition of lean tissue (Rivera‐Ferre et al., 2005), which in turn are determined by its unique traditional open‐air production system (Lopez‐Bote, 1998) and its genetic features (Alves et al., 2003, Fabuel et al., 2004, Ollivier, 2009).

The measurement of MB content is not a straightforward technique (Hornsey, 1956) and it might be advisable to use molecular information to include MB as a selection goal in a breeding program. Whole transcriptome sequencing of divergent individuals for a particular trait allows the identification of candidate genes for these traits and, at the same time, a better understanding of the gene networks and biological pathways underlying the concerned trait. Analyses of changes in the transcriptome between divergent individuals for a particular trait such as intramuscular fat, tenderness, or feed efficiency through RNA‐seq have been carried out in different studies (Fernández‐Barroso et al., 2020b; Muñoz et al., 2018; Vigors et al., 2019; Zappaterra et al., 2020). However, to our knowledge, this work is novel being the first transcriptomic study of porcine muscle divergent in estimated breeding values (EBVs) for myoglobin content.

In the current study we sequenced the transcriptome of longissimus dorsi (LD) muscle in divergent Iberian pigs for MB content. Hence, the aims of this study were: (i) to identify and quantify differentially expressed genes (DEGs) between divergent groups; (ii) carry out in silico functional analyses for a better comprehension of the biological pathways that could be involved in the differences in MB content; and (iii) identify the transcriptional regulatory factors influencing the observed gene expression profiles.

## MATERIALS AND METHODS

### Animal material and phenotypic data

Animal handling was carried out according to the regulations of the Spanish Policy for Animal Protection RD 53/2013, which meets the European Union Directive 2010/63/EU about the protection of animals used in research. Protocols were assessed and approved by the INIA Committee of Ethics in Animal Research, which is the named Institutional Animal Care and Use Committee for the INIA.

The animal material used in the present study was obtained from castrated males belonging to a closed commercial population of Iberian pigs. The animals were fed under a restricted feeding regime until they reached 100 kg of body weight and were subsequently fattened in an open‐air free‐range system until slaughter, with an approximate age of 17 months and 165 kg of final body weight.

After slaughter, LD samples were removed from the carcass of 828 animals and a central muscle section of approximately 80 g was separated of each loin for MB content measurement. The muscle portions were vacuum packed in nylon/polyethylene bags, and then the samples were introduced in liquid N_2_ for approximately 20 s, before storing at −20°C until determination of the MB content. After that, the samples were thawed and MB was measured as mg myoglobin/g muscle as described in Fernández‐Barroso et al. (2020a) following Horsney (1956) with modifications from Alberti et al. (2005). The MB mean was 1.77 mg/g (SD = 0.31).

The following mixed model was used to estimate the breeding values (EBVs) for MB content:

y=Xb+Za+Wsm+e

where **y** is the vector of MB values corresponding to each animal; **b** represents the vectors of systematic effects, in which the percentage of intramuscular fat percentage (IMF), the slaughter age and the average weight of the two loins for each individual were fitted as covariates; **a** is the vector of the additive genetic effects (EBVs) distributed as *N* (0, ${A\sigma }_{u}^{2}$), where ***A*** is the numerator of the kinship matrix that allows for the adjustment of the data taking into account the pedigree information; **sm** is the vector of the environmental random effects caused by the combined fattening‐slaughter batches (24 levels), and **e** is the vector including the residual effects. **X**, **Z**, and **W** are the incidence matrices. EBVs were estimated using the pest 4.1 (Groeneveld et al., 1999) and vce‐6 programs (Groeneveld et al., 2010).

A total of 12 pigs with the most extreme EBVs for MB were selected, six per each group, avoiding full siblings. The most extreme EBVs animals belonged to the same season, therefore, some possible environmental effects associated with the annual environmental differences were reduced. The mean phenotypic values of MB content were 2.48 g/kg (SD = 0.07) for the six individuals that showed the highest EBVs (High MB group) and 1.39 g/kg (SD = 0.15) for the six with the lowest EBVs (Low MB group); the corresponding EBVs averages were 0.18 (SD = 0.02) and −0.20 (SD = 0.04) respectively.

### Transcriptomic analyses

#### RNA extraction, library preparation, and sequencing

The loin samples collected after slaughter were introduced in cryogenic tubes, frozen in liquid nitrogen and stored at −80°C until analysis. The RiboPure™ High‐Quality RNA Purification kit (Ambion) was used to extract total RNA, following the manufacturer's recommendations. NanoDrop equipment (NanoDrop Technologies) was used to quantify the RNA and Agilent 2100 Bioanalyzer device (Agilent Technologies) was used to measure RNA integrity (RNA integrity number). The values obtained for all the samples were higher than 8.

NEBNext^®^ Ultra™ RNA Library Prep Kit (Illumina) was used to build the paired end libraries for each sample. Novaseq 6000 sequence analyzer (Illumina Inc) to carry out multiplex sequencing of the libraries, with four samples per lane at Novogene (Novogene UK Company Limited), according to the manufacturer's instructions. Pair end reads of 150 bp were generated. The raw sequence data of the 12 animals has been deposited in the Gene Expression Omnibus database with the accession number: GSE178915.

#### Bioinformatics analyses

Quality of raw sequencing data was assessed with FastQC (Babraham Bioinformatics, http://www.bioinformatics.babraham.ac.uk/projects/fastqc/). Quality was measured according to sequence read lengths and base‐coverage, nucleotide contributions and base ambiguities, quality scores, and over‐represented sequences. All the samples passed the quality control parameters: same length, 100% coverage in all bases, 25% of A, T, G, and C nucleotide contributions, 50% GC on base content and <0.1% of overrepresented sequences. TrimGalore (Babraham Bioinformatics, http://www.bioinformatics.babraham.ac.uk/projects/trim_galore/) was used to trim the raw sequences through removing the sequencing adaptor and poly A and T tails, setting default values (6 bp stringency) and keeping paired‐end reads when both pairs were longer than 40 bp. hisat2 (Kim et al., 2019) was used to map the filtered reads against the pig reference genome (sscrofa11.1). After that, htseq‐counts software (Anders et al., 2015) was employed to obtain raw counts for the genes and transcripts and to construct the read counts matrix. Then, differential expression analyses were carried out using the deseq2 package (Love et al., 2014) in r environment (Team, 2015). Genes and transcripts were considered as differentially expressed (DEGs) when the log_2_ fold change (log_2_ FC) of the expression differences between the High MB and Low MB groups were lower than −0.58 and higher than 0.58 and with a *p*‐value <0.05. The false discovery rate was adjusted keeping those DEGs with a *q*‐value <0.10. In addition, unsupervised hierarchical clustering analyses with the whole expression profile and considering just the DEG expression profile per each individual sample were carried out.

#### Gene functional classification, network, and pathway analyses

The functionality of the DEGs was analyzed using gene ontology (GO) information. The biological interpretation of the data was performed using FatiGO browser from babelomics 5 (Babelomics 5, http://babelomics.bioinfo.cipf.es/). string tools v11.0 (Szklarczyk et al., 2017) was used to study the potential interactions between the proteins codified by the DEGs and clustering through the Markov Cluster Algorithm.

The bioinformatic tool, Ingenuity Pathway Analysis (IPA, Ingenuity Systems) was used to identify and characterize biological functions, gene networks, canonical pathways, and transcription regulatory factors affected by the DEGs. This software assesses the significant association between the data set of DEGs and canonical pathways. In addition, the biological relationships between genes are represented with networks graphs, which were built with the set of genes using the records harbored in the Ingenuity Pathways Knowledge Base. Potential regulators of differential gene expression were also identified using the tools ‘upstream regulators’ and ‘causal networks’. These tools analyze whether the potential transcriptional factors and upstream regulators contained in the Ingenuity Knowledge Base repository activate or inhibit the differential gene expression pattern through estimating a z‐score. The z‐score statistically measures the significance between the regulator and its potential targets as well as the direction among them (Krämer et al., 2014).

### RNA‐seq results validation by qPCR

To perform the technical validation of the RNA‐seq experiment, we used RNA samples from the same 12 pigs analyzed in the RNA‐seq study. We carried out the validation by measuring the expression of 11 genes with qPCR; seven genes differentially expressed between the High and Low group (*CD209*, *HBA1*, *PLA2G10*, *ZSCAN31*, *EFEMP1*, *LGALS3*, and *MX2*) and four of them not differentially expressed (*ATP6*, *DGAT2*, *ELOVL5*, and *SCD*).

Firstly, first‐strand cDNA synthesis was carried out using Superscript II (Invitrogen, Life Technologies) and random hexamers, in a total volume of 20 µl using 1 µg of total RNA, according to the manufacturer's instructions. Primer pairs used for quantification were designed using Primer‐Blast (NCBI, https://www.ncbi.nlm.nih.gov/tools/primer‐blast/) from the available GENBANK and/or Ensembl sequences, covering different exons to assure the amplification of the cDNA. These primer sequences and amplicon lengths is shown in Table S1. Next, a standard PCR on cDNA was performed for each primer to verify amplicon sizes. Then, the quantification was carried out with SYBR Green Mix (Roche) in a lightcycler480 (Roche) and data analysis was performed with lightcycler480 SW1.5 software (Roche). Three technical replicates were run per each sample and dissociation curves were obtained to confirm the specific amplification of each gene. Four cDNA dilutions were carried out to build a standard curve and estimate the PCR efficiency. Statistical analysis was performed using the mean crossing point values (*C*_p_), which is the PCR cycle number when the sample's reaction curve cuts the threshold line. The stability of the endogenous genes *ACTB* and *B2M* was calculated with genorm (Vandesompele et al., 2002). The relative quantities of DEGs were divided by the geometric means of the two reference genes (as a normalization factor). The statistical differences between qPCR mean values of High and Low groups were analyzed by means of Student's *t*‐test. For the technical validation, we calculated the Pearson correlation between the expression values from RNA‐seq and from qPCR, also the concordance correlation coefficient (CCC) between the fold change values from the two techniques was estimated.

In addition, a primer pair was designed to measure the expression of the *MB* gene using the sequence deposited in the Genbank database with the accession number NM_214236.1 (Table S1). Statistical differences between the mean expression values of High and Low groups were also analyzed using Student's *t*‐test.

## RESULTS

### Characterization of LD transcriptome and differential expression analyses

The LD transcriptome of the 12 selected pigs was characterized through RNA‐seq technique. We obtained a total of 1498 million raw paired end reads. After the trimming and filtering processes, 1497 million reads remained. All samples passed the quality control and 91.80%–94.07% of the reads were mapped to the porcine reference genome (sscrofa11.1; Table S2).

**FIGURE 1:**
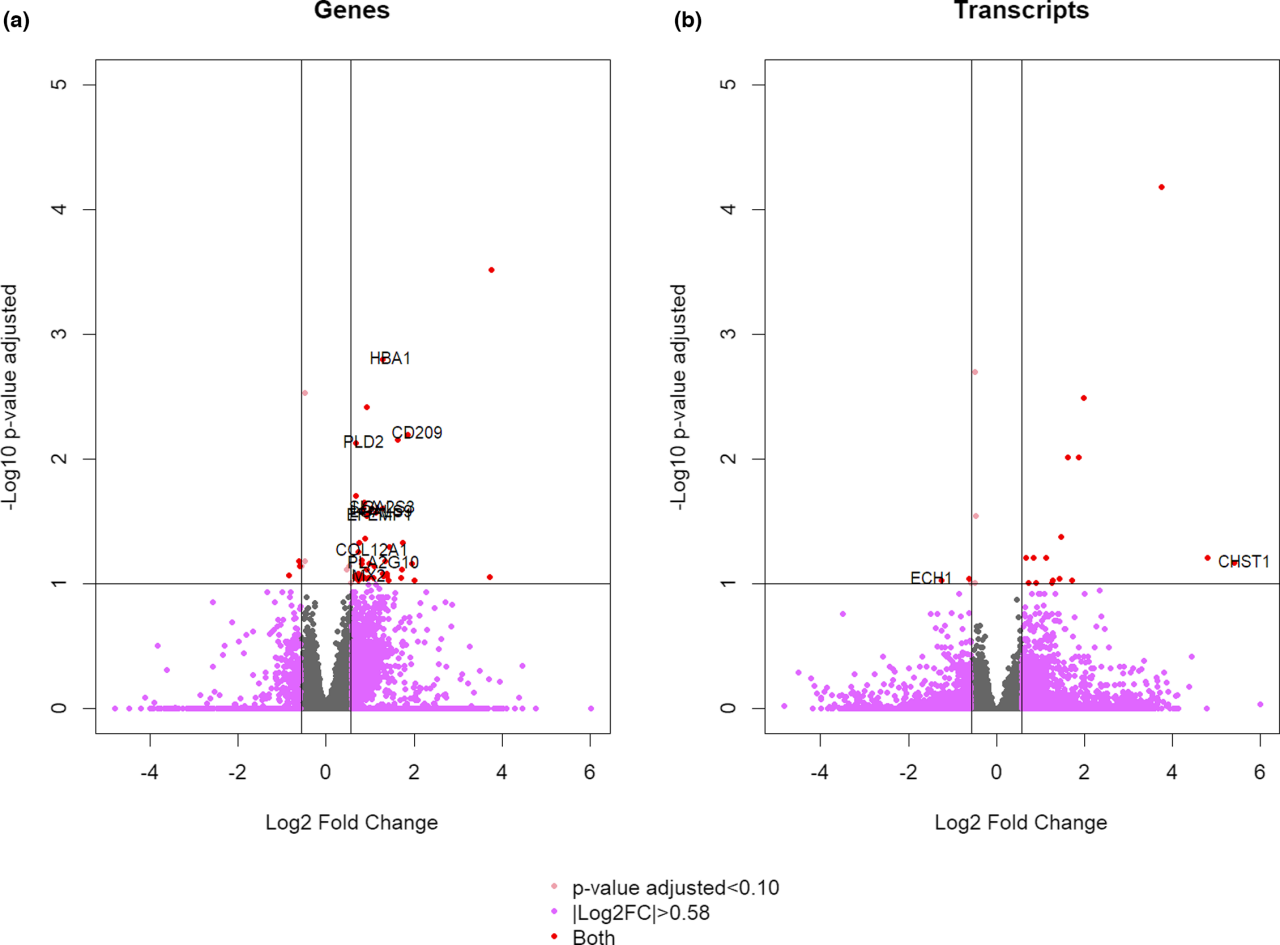
Volcano plot of differentially expressed genes (DEGs) and transcripts of High myoglobin group vs. Low myoglobin group. Red dots indicate DEGs with *q*‐value <0.10 and |Log_2_ fold change| > 0.58. Grey dots are non‐significant genes

A total of 16,746 out of 22,452 genes annotated in the reference genome were detected as expressed in our samples and a total of 17,226 transcripts were expressed. In addition to the genes complying the established filters (|log_2_ FC| > 0.58 and *q* < 0.10), the transcripts fulfilling these filters and belonged to genes not included in the previous dataset were considered as DEGs. The volcano plot (Figure 1a,b) graphically represents the expressed genes and transcripts identified as differentially expressed. Finally, 57 DEGs were identified in the High and Low MB groups, 53 DEGs were upregulated in the High group (log_2_ FC ≥ 0.58) while four were upregulated in the Low group (log_2_ FC ≤ −0.58; Table S3). The unsupervised hierarchical clustering using the expression profile of all the genes was not being able of separating samples in the two analyzed groups (Figure S1a); however, when the expression profile of DEGs was taken into account, this analysis did properly split the samples in the High and Low MB groups (Figure S1b).

Regarding the expression values, log_2_ FC ranged from −1.26 to 5.42, being the genes with the highest expression differences between the divergent groups *carbohydrate sulfotransferase 1 (CHST1*, log_2_ FC = 5.42, *p* = 2.16 × 10^−5^, upregulated in the High group) and *enoyl*‐*CoA hydratase 1 (ECH1*, log_2_ FC = −1.26, *p* = 1.33 × 10^−4^, upregulated in the Low group; Table S3). Table 1 shows a list of DEGs chosen for their key functions, which would be associated with the biological pathways of the MB content.

**TABLE 1:** Log_2_ fold change, basemean expression value in the High and Low myoglobin groups, *p*‐value, and *q*‐value, corresponding to the most relevant differentially expressed genes (DEGs)

Gene	Log_2_ FC	High	Low	*p*‐Value	*q*‐Value
*ECH1*	−1.23	27.56	65.85	1.33 × 10^−04^	0.093
*PLD2*	0.68	247.10	154.71	9.13 × 10^−06^	0.007
*COL12A1*	0.73	286.49	172.22	8.69 × 10^−05^	0.055
*MX2*	0.82	185.83	104.97	6.74 × 10^−04^	0.089
*SLA‐1*	0.85	14003.78	7759.99	1.00 × 10^−05^	0.025
*EFEMP1*	0.93	570.37	299.29	4.36 × 10^−05^	0.029
*PLA2G10*	0.97	91.26	46.57	3.15 × 10^−04^	0.069
*LGALS9*	0.98	175.16	88.68	3.82 × 10^−05^	0.027
*LGALS3*	1.03	371.36	181.74	3.22 × 10^−05^	0.025
*HBA1*	1.29	181.23	73.37	4.97 × 10^−07^	0.002
*SLA‐7*	1.37	327.64	126.65	3.47 × 10^−04^	0.085
*CD209*	1.86	342.77	94.59	3.44 × 10^−06^	0.006
*CHST1*	5.42	7.74	0.181	2.16 × 10^−05^	0.068

Note

High and Low: basemean expression value calculated from the coefficients (*β*_0_ and *β*_1_) estimated by the generalized linear model fit in DESeq2 (Love et al., 2014).

### Gene functional analyses

FatiGO was employed to perform GO enrichment analyses, which recognized 24 GO biological processes (GO_BP_) and one GO_SLIM_ (cut‐down versions of the GO ontologies containing a subset of the terms in GO) enriched in DEGs (Table 2). There was an enrichment of DEGs in the processes involved in the metabolism of prostaglandins. Functional enrichment analyses also showed biological processes involved in the metabolism of eicosanoids, oxidation status, fatty acid transport, reactive oxygen species metabolism, T Cell proliferation, cytoskeleton organization and connective tissue.

**TABLE 2:** List of significantly overrepresented gene ontology (GO) terms related to myoglobin content on differentially expressed genes (DEGs) using FatiGO

Term	Genes	Adjusted *p*‐value
GO_BP_		
Prostaglandin secretion (GO:0032310)	*P2RX7*, *PLA2G10*	0.026
Positive regulation of icosanoid secretion (GO:0032305)	*P2RX7*, *PLA2G10*	0.026
Regulation of prostaglandin secretion (GO:0032306)	*P2RX7*, *PLA2G10*	0.026
Positive regulation of prostaglandin secretion (GO:0032308)	*P2RX7*, *PLA2G10*	0.026
Mitochondrial depolarization (GO:0051882)	*P2RX7*, *IFI6*	0.026
Regulation of mitochondrial depolarization (GO:0051900)	*P2RX7*, *IFI6*	0.026
Prostaglandin transport (GO:0015732)	*P2RX7*, *PLA2G10*	0.026
Positive regulation of fatty acid transport (GO:2000193)	*P2RX7*, *PLA2G10*	0.028
Regulation of icosanoid secretion (GO:0032303)	*P2RX7*, *PLA2G10*	0.034
Monocarboxylic acid transport (GO:0015718)	*P2RX7*, *PLA2G10*, *SLC16A7*	0.037
Positive regulation of organic acid transport (GO:0032892)	*P2RX7*, *PLA2G10*	0.037
Regulation of fatty acid transport (GO:2000191)	*P2RX7*, *PLA2G10*	0.040
Reactive oxygen species metabolic process (GO:0072593)	*P2RX7*, *PRCP*, *HBA1*	0.040
T cell proliferation (GO:0042098)	*P2RX7*, *CD209*, *LGALS3*	0.040
Cartilage development (GO:0051216)	*EFEMP1*, *SCIN*, *MGP*	0.040
Cortical actin cytoskeleton organization (GO:0030866)	*EPB41L3*, *EPB41L1*	0.040
Positive regulation of ion transport (GO:0043270)	*P2RX7*, *PLA2G10*, *LGALS3*	0.040
Positive regulation of cytoskeleton organization (GO:0051495)	*P2RX7*, *SCIN*, *FES*	0.040
Cortical cytoskeleton organization (GO:0030865)	*EPB41L3*, *EPB41L1*	0.043
Icosanoid secretion (GO:0032309)	*P2RX7*, *PLA2G10*	0.047
Icosanoid transport (GO:0071715)	*P2RX7*, *PLA2G10*	0.047
Fatty acid derivative transport (GO:1901571)	*P2RX7*, *PLA2G10*	0.047
Connective tissue development (GO:0061448)	*EFEMP1*, *SCIN*, *MGP*	0.047
Phosphatidylglycerol metabolic process (GO:0046471)	*PLA2G10*, *PLD2*	0.047
GO_slim_		
Proteinaceous extracellular matrix (GO:0005578)	*EFEMP1*, *MGP*, *FBLN1*	0.01

**FIGURE 2:**
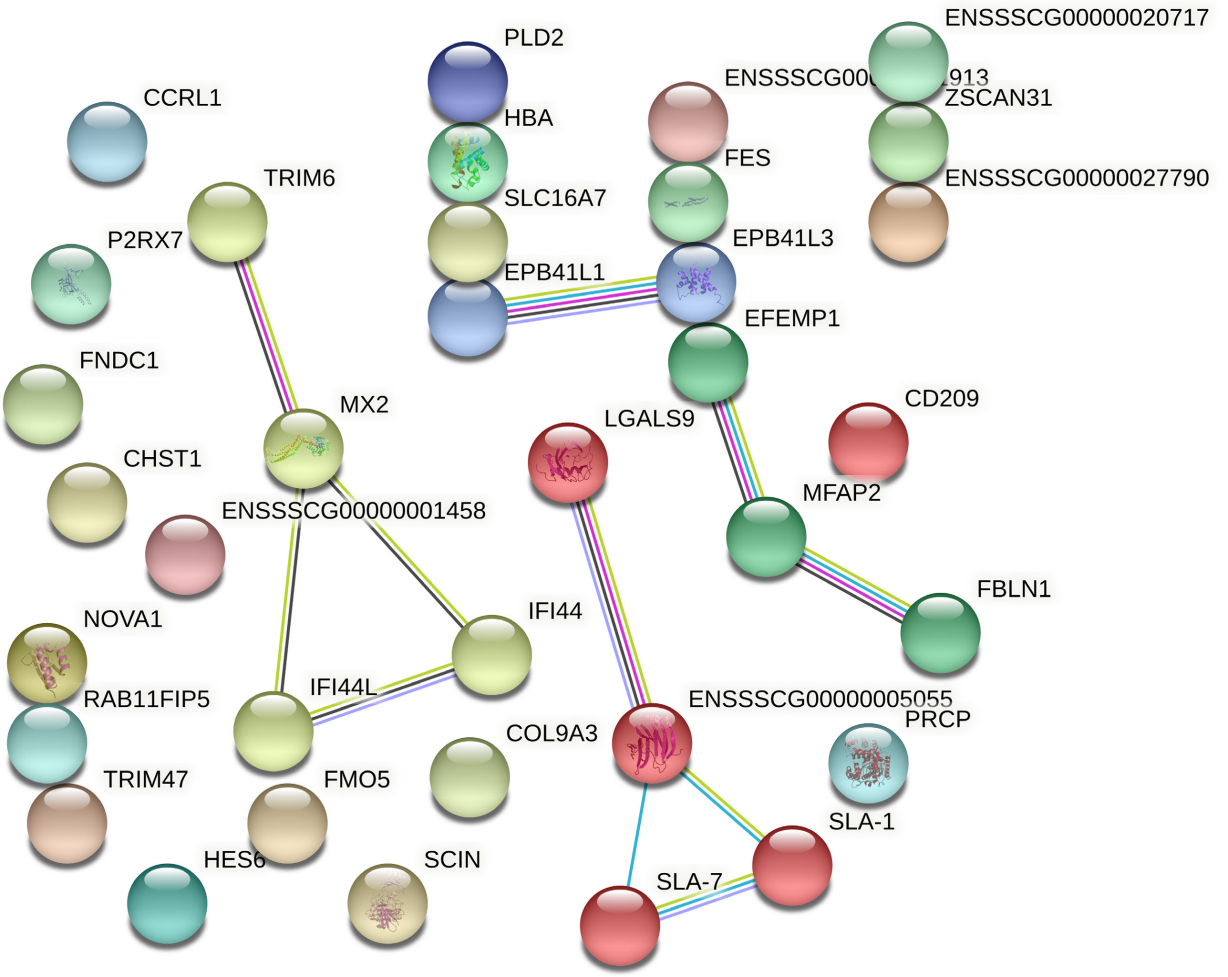
Network of protein‐protein interactions predicted with string database. Same color nodes sharing multiple edges are grouped in the same cluster

Figure 2 shows the results obtained from STRING, which identified networks of protein–protein interactions codified by annotated DEGs. We found four differentiated clusters comprising proteins codified by DEGs; these proteins are all upregulated in the High MB group. Cluster 1 is constituted by LGALS9, ENSSSCG00000005055 (LGALS3), SLA‐1, and SLA‐7 involved in cellular and molecular recognition; moreover, LGALS3 is involved in immune metabolism GO_BP_ (Table 2). Cluster 2 is constituted by TRIM6, MX2, IFI44, and IFI44L involved in defense against virus. Cluster 3 is constituted by EPB41L1 and EPB41L3, associated with actin binding and actomyosin and cytoskeleton structure organization. Lastly, cluster 4 is constituted by EFEMP1, MFAP2 and FBLN1, associated with cell function, cell adhesion, and degradation of the extracellular matrix.

**FIGURE 3:**
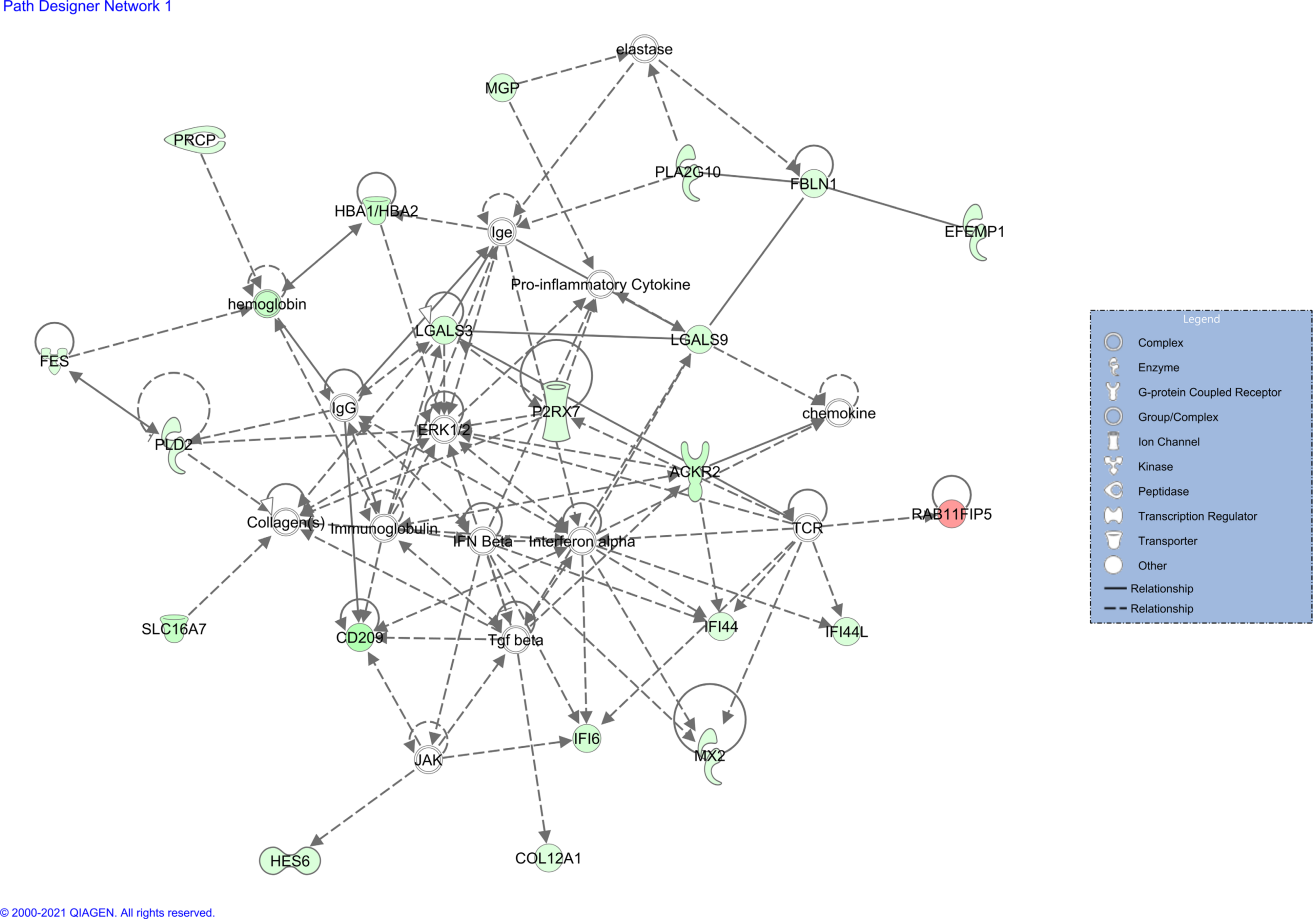
Gene network 1: dermatological diseases and conditions, immunological disease, organismal injury and abnormalities. Genes upregulated and downregulated in the High myoglobin group are represented in green and red colors respectively

**FIGURE 4:**
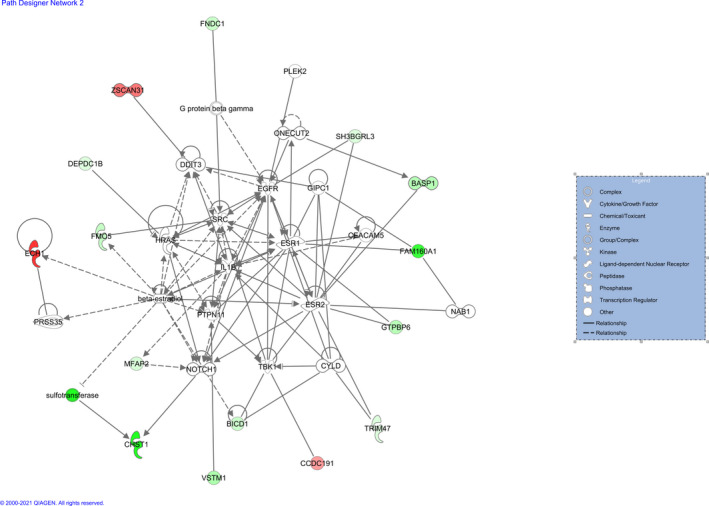
Gene network 2: cell cycle, gene expression, lipid metabolism. Genes upregulated and downregulated in the High myoglobin group are represented in green and red colors respectively

The additional functional analysis performed with IPA software revealed four networks enriched in DEGs (Table 3). IPA assigns a network score in concordance to the size of the network and the number of target genes involved. This score is estimated as the negative logarithm of the *p*‐value calculated by Fisher's exact test. The most relevant function represented in network 1 was *immunological disease* (Figure 3), in network 2 *lipid metabolism* (Figure 4), in network 3 *hematological system* (Figure S2) and in network 4 *lipid metabolism* (Figure S3).

**TABLE 3:** Relevant enriched networks and functions related to myoglobin content identified in the set of differentially expressed genes between the High and Low groups identified by IPA software

ID	Molecules in network	Score	Focus molecules	Functions
1	**ACKR2, CD209**, chemokine, **COL12A1**, collagen(s), **EFEMP1**, elastase, ERK1/2, **FBLN1, FES**, **HBA1/HBA2**, hemoglobin, HES6, **IFI44, IFI44L, IFI6**, IFN β, Ige, IgG, immunoglobulin, interferon α, JAK, **LGALS3, LGALS9, MGP, MX2, P2RX7, PLA2G10, PLD2, PRCP**, pro‐inflammatory cytokine, **RAB11FIP5, SLC16A7**, TCR, TGF β	54	21	Dermatological diseases and conditions, immunological disease, organismal injury and abnormalities
2	**BASP1, β‐estradiol, BICD1, CCDC191, CEACAM5, CHST1**, chymotrypsin, CYLD, D‐glucose, DDIT3, **DEPDC1B, ECH1**, EGFR, ESR1, ESR2, **FAM160A1, FMO5**, FNDC1, G protein βγ, GIPC1, **GTPBP6**, HRAS, IL1B, **MFAP2**, NAB1, NOTCH1, ONECUT2, PLEK2, PRSS35, PTPN11, **SH3BGRL3**, SRC, sulfotransferase, TBK1, TRIM47, **VSTM1**, **ZSCAN31**	35	15	Cell cycle, gene expression, lipid metabolism
3	**ACKR4**, actin, ADGRG3, Akt, anti‐inflammatory cytokine, C1GALT1C1, caspase, CCL27, Cyb5r3, cysteinyl‐leukotriene, **EPB41L1, EPB41L3**, ERK, estrogen receptor, Histone h3, HTR1B, ICAM2, ICAM3, JAM2, lewis Y, mannan, mannose, **MAPK1**, NFκB (complex), NLRC4, NOVA1, NRG3, P38 MAPK, Pak, **RPRM, SCIN**, SPINK7, STMN4, **TRIM34**, Vegf	14	7	Cell‐to‐cell signaling and interaction, hematological system development and function, immune cell trafficking
4	ANGPTL3, ANGPTL4, ANGPTL8, APOA5, **GPIHBP1**, immunoglobulin, LPL, oleic acid	2	1	Cardiovascular disease, lipid metabolism, small molecule biochemistry

#### Canonical pathways analysis

We carried out a functional interpretation of the global gene expression differences using IPA canonical pathway analysis. Regarding the dataset of 57 DEGs, a total of four canonical pathways were significantly enriched (*p* < 0.05, Table 4). The most significant pathway was *phospholipases* (*p* = 0.007) and the other three relevant pathways were *antioxidant action of vitamin C*, *choline biosynthesis III*, and *inflammasome pathway*. All the molecules included in these pathways are upregulated in the High MB group. Nevertheless, the functional analysis did not report any pathway with assigned z‐score, therefore none of them were predicted for an overall activation or inhibition in the High or Low MB groups.

**TABLE 4:** List of significant pathways (*p*‐value <0.05) identified in the set of differentially expressed genes according to the High and Low myoglobin groups identified by Ingenuity Pathway Analysis (IPA) software

Canonical pathways	*p*‐Value	Ratio	Molecules
Phospholipases	0.007	0.031	PLA2G10, PLD2
Antioxidant action of vitamin C	0.019	0.018	PLA2G10, PLD2
Choline biosynthesis III	0.028	0.067	PLD2
Inflammasome pathway	0.038	0.050	P2RX7

Note

Ratio: number of differentially expressed genes in a pathway divided by the number of genes comprised in the same pathway.

#### Transcription regulatory factors

The IPA upstream analysis and regulator effect tools were used to determine the potential transcription regulatory factors of DEGs involved in molecular processes, which may explain the differential expression observed between the High and Low MB groups. In this study, a total of 156 transcriptional regulators were found (*p* < 0.05, Table S4). The direction of the activation state of five regulators was statistically predicted (*z*‐score >2 or *z*‐score <−2, Table 5). PRL, IFNG, and IRF7 were predicted as activated in the High MB group (*z*‐score >2) while IL1RN and MAPK1 were activated in the Low MB group (*z*‐score <−2). In addition to this, SPI1 (*p* = 2.63 × 10^−4^, Table S4) presented a positive *z*‐score (1.98), indicating a trend for activation in the High MB group.

**TABLE 5:** List of significant upstream regulators identified in the set of differentially expressed genes according to the High and Low myoglobin (MB) groups (*p*‐value < 0.05 and *z*‐score >2 or <−2)

Upstream regulator	Molecule type	PAS	*z*‐Score	*p*‐Value of overlap	Molecules in dataset
PRL	Cytokine	High MB	2.22	2.35 × 10^−04^	IFI44, IFI44L, IFI6, MGP, MX2
IFNG	Cytokine	High MB	2.04	7.49 × 10^−03^	CD209, FBLN1, IFI44, IFI44L, IFI6, LGALS3, LGALS9, MX2
IRF7	Transcription regulator	High MB	2.00	3.61 × 10^−04^	IFI44, IFI44L, IFI6, MX2
IL1RN	Cytokine	Low MB	−2.24	5.78 × 10^−06^	IFI44, IFI44L, IFI6, LGALS9, MX2
MAPK1	Kinase	Low MB	−2.65	9.72 × 10^−06^	FES, HBA1/HBA2, IFI44, IFI6, LGALS3, MX2, TRIM34

Abbreviation: PAS, predicted activation ratio.

**FIGURE 5:**
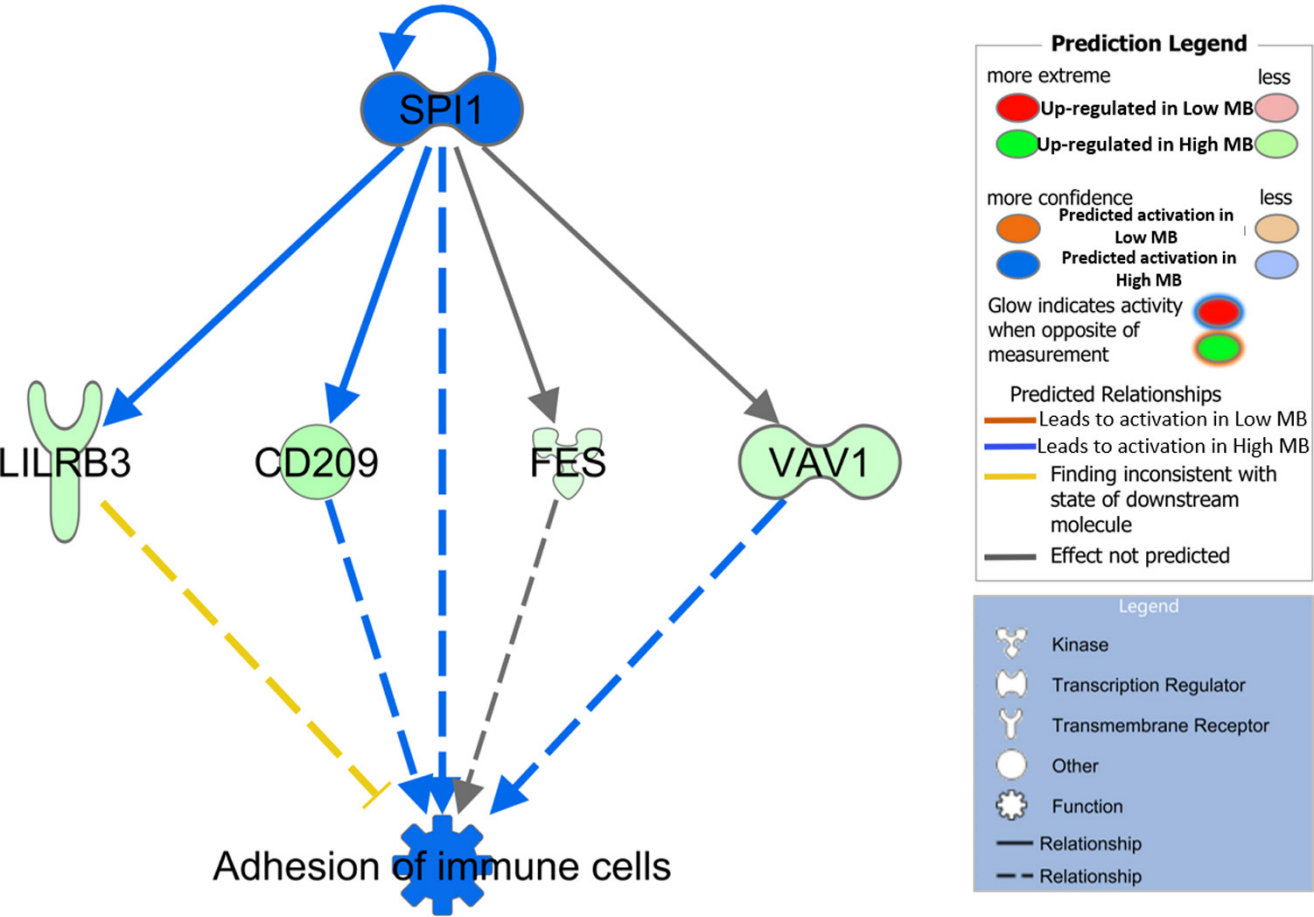
Master regulator effects network predicted in Iberian pigs fattened in an open‐air free‐range system. In the upper tier is SPI1 (predicted to be activated, blue color). In the middle, there are the genes whose expression changes in response to the activation of SPI1 (green upregulated for the High myoglobin group). Dashed lines between SPI1 and differentially expressed genes represent the interactions, predicted to be activated (blue lines) or predicted to be inhibited (orange lines). In the lower tier, the expected phenotypic activate function (adhesion of immune cells, blue color) is shown

Furthermore, a set of master regulators were statistically predicted (Table 6), where four were activated in the High MB group (*z*‐score >2; SPI1, PRL, IRF7, IFNA2) and another four in the Low MB group (MAPK1, IL1RN, MECP2, Hnf3). A complementary functional analysis was carried out with IPA considering those genes with a *p*‐adjusted‐value <0.20. With this data set, a regulatory effect network (Figure 5) was predicted, representing a causal hypothesis to interpret the potential mechanism of the master regulator (SPI1) in the expression of some DEGs.

**TABLE 6:** Ingenuity Pathway Analysis (IPA)

Master regulator	Molecule type	Participating regulators	PAS	*z*‐Score	*p*‐Value of overlap	Target molecules in dataset
MAPK1	Kinase	MAPK1	Low MB	−2.65	2.52 × 10^−06^	FES, HBA1/HBA2, IFI44, IFI6, LGALS3, MX2, TRIM34
IL1RN	Cytokine	IL1RN	Low MB	−2.24	5.16 × 10^−06^	IFI44, IFI44L, IFI6, LGALS9, MX2
PRL	Cytokine	PRL	High MB	2.24	1.99 × 10^−04^	IFI44, IFI44L, IFI6, MGP, MX2
IRF7	Transcription regulator	IRF7	High MB	2.00	3.15 × 10^−04^	IFI44, IFI44L, IFI6, MX2
MECP2	Transcription regulator	MECP2, SPI1	Low MB	−2.24	3.56 × 10^−04^	CD209, HBA1/HBA2, IFI44, IFI44L, IFI6
IFNA2	Cytokine	IFNA2	High MB	2.00	5.91 × 10^−04^	IFI44, IFI44L, IFI6, MX2
SPI1	Transcription regulator	SPI1	High MB	2.00	6.14 × 10^−04^	CD209, IFI44, IFI44L, IFI6
Hnf3	Group	Estrogen receptor, FOXA1, FOXA2, FOXA3, Hnf3, IRF3, STAT5B	Low MB	−2.65	1.68 × 10^−03^	COL12A1, FBLN1, IFI44, IFI44L, IFI6, MGP, RPRM

Note

List of significant master regulators (*p* < 0.05) with assigned *z*‐score identified in the set of differentially expressed genes according to High and Low myoglobin (MB) groups.

Abbreviation: PAS, predicted activation ratio.

#### RNA‐seq validation by qPCR

To validate the RNA‐seq results, we calculated the Pearson correlation with the quantification of the expression of eleven genes using qPCR in the same 12 samples. Likewise, the corresponding *p*‐values and CCC were obtained (Table 7). Seven genes showed a correlation coefficient higher than 0.75 and all of them showed a significant *p*‐value (<0.05). The CCC was equal to 0.881 (CI95%: 0.696–0.956), suggesting a substantial concordance between RNA‐seq and qPCR expression values (Miron et al., 2006). *CD209* and *ZSCAN31* genes showed the highest and the lowest concordance between methods respectively.

**TABLE 7:** Technical validation of RNA‐seq results by quantitative PCR (qPCR)

Gene	qPCR	qPCR	Expression type	RNA‐seq	RNA‐seq	*r* ^2^	*r* ^2^ *p*‐Value	CCC
	Log_2_ FC	*p*‐Value		Log_2_ FC	*q*‐value			
*CD209*	1.20	0.034	H>L	1.86	0.006	0.990	6.39 × 10^−10^	0.880
*SCD*	0.28	0.383	NO DE	0.54	0.460	0.969	2.25 × 10^−7^	
*DGAT2*	0.54	0.196	NO DE	0.85	0.145	0.902	5.91 × 10^−5^	
*ELOVL5*	0.21	0.317	NO DE	0.65	0.473	0.861	3.24 × 10^−4^	
*HBA1*	0.33	0.079	H>L	1.29	0.016	0.664	0.019	
*ATP6*	0.01	0.282	NO DE	0.09	1	0.747	0.005	
*PLA2G10*	0.96	0.119	H>L	0.97	0.069	0.623	0.041	
*ZSCAN31*	−0.83	0.010	L>H	−0.84	0.085	0.590	0.041	
*EFEMP1*	0.47	0.027	H>L	0.93	4.36 × 10^−5^	0.727	0.007	
*LGALS3*	0.54	0.057	H>L	1.02	0.025	0.877	1.78 × 10^−4^	
*MX2*	0.42	0.036	H>L	0.83	0.089	0.810	0.001	
*MB1*	0.202	0.166	–	–	–	–	–	–

Notes

qPCR *p*‐value corresponds to the *t*’ student test analyzing the expression differences between the groups H and L; RNA‐seq *q*‐value corresponds to the RNA‐seq differential expression analyses and *r* ^2^ *p*‐value corresponds to the Pearson correlation analyses. Fold Change values (FC), Pearson correlations (*r* ^2^) and concordance correlation coefficient (CCC) between expression values obtained from both techniques.

Abbreviations: H>L, higher expression in High myoglobin (MB) group than in Low MB; L>H, higher expression in Low MB group than in High MB; NO DE, no differentially expressed in RNA‐seq experiment.

Lastly, a higher mean expression value measured by qPCR for *MB* gene was observed in the High (0.733 ± 0.209) than in the Low group (0.638 ± 0.202), however, no statistical differences were observed between these groups (*p* = 0.166).

## DISCUSSION

The red color of the meat is associated with consumer preferences and, at the same time, is related to its content in myoglobin. In the current study, transcriptome analysis between divergent Iberian pigs for breeding values of myoglobin content showed 57 DEGs and a set of functional pathways and protein networks in which they are involved. These results provide more insight into the mechanisms of the processes underlying this trait.

The first expected result would be that the gene encoding myoglobin was differentially expressed. Nevertheless, this gene was not annotated in the version of the pig reference genome used in the present study (sscrofa11.1). To check if the *MB* gene could be differentially expressed, we measured its expression by qPCR and, although we observed higher expression values in the High than in the Low group , no significant differences between groups were observed. Yu et al. (2017) observed an increase of MB content in LD muscle with the age on Duroc × Landrace × Yorkshire pigs and a similar trend for *MB* gene expression; however, the gene expression pattern was not statistically significant. Other authors did find statistically significant differences in MB gene expression and protein content when different muscles (Kim et al., 2004) or diet supplementation were compared (Li et al., 2013). The results observed in the present study could suggest that the MB protein content could be regulated not only by transcriptional mechanisms but also by post‐transcriptional ones.

*Hemoglobin subunit alpha 1 (HBA1)* encoding α‐globin, which is a component of hemoglobin responsible for carrying oxygen to cells and tissues all over the body, is one of the upregulated DEG in the High MB group. The functional analysis revealed that *HBA1* play a role in the metabolic process of reactive oxygen species (ROS; GO:0072593, Table 2). The function and regulation of non‐erythrocyte hemoglobin is not fully understood; however, oxidative stress seems to be associated with higher hemoglobin expression in cells other than erythrocytes (Liu et al., 2011). In addition, the myoglobin and hemoglobin content in striated muscles is correlated in several species, including pigs (O'Brien et al., 1992), and these authors associated the hemoglobin content with a greater aerobic capacity of the tissue as well as its blood flow, and the myoglobin content with high physical activity. Therefore, a higher expression of hemoglobin and myoglobin could protect against oxidative stress.

Several of the DEGs found in the current study such as *phospholipase A2 group X* (*PLA2G10*) and *phospholipase D2* (*PLD2*), both upregulated in the High MB group, and *ECH1*, which is upregulated in the Low MB group, are involved in lipid metabolism. The *PLA2G10* gene encodes for a lipolytic enzyme, which plays a role in lipid pathways such as the hydrolysis of cell membrane phospholipids and the release of free fatty acids and lysophospholipids (Murakami et al., 2020; Vadas & Pruzanski, 1986). According to the functional enrichment analysis, this gene is involved in the regulation of prostaglandin secretion and transport (GO:0032306, GO:0032308, GO:0015732), fatty acid transport (GO:2000193, GO:2000191), and regulation of eicosanoid secretion (GO:0032303, GO:0032305; Table 2). The enzyme has a role maintaining membrane phospholipid homeostasis (Sun et al., 2010), as well as its function is important in inflammation since it releases arachidonic acid, a precursor of eicosanoids (Hanasaki et al., 2002). It has been suggested that PLA2G10 protein could be involved in immune functions, such as an anti‐inflammatory phenotype, and the enzyme also acts as an important defense mechanism against intestinal parasites and virus, with a role in adaptive and innate immune responses (Murakami et al., 2020). While PLA2G10 has been used as an inflammatory marker, MB has been used as tissue injury marker (Tartibian et al., 2011) because high levels are related to a high vulnerability of the membrane (Driessen‐Kletter et al., 1990). Therefore, this gene would perform a double key function all at once: lipid and immune metabolism. Several authors (Batista‐Gonzalez et al., 2020; Gianfrancesco et al., 2019; Hubler & Kennedy, 2016) described that lipid metabolism plays a role in the regulation of immune cell activation, highlighting the connection between both functions.

The *PLD2* gene codifies an enzyme that plays a pivotal role in the regulation of cell function and cell fate (Liscovitch et al., 2000). The functional analysis revealed that this gene is involved in the *choline biosynthesis III* pathway (Table 4). In the same way, Yang et al. (2004) described that PLD2 hydrolyzes phosphatidylcholine from the cell membrane generating phosphatidic acid, which is a lipid messenger that mediates signaling functions. Phosphatidic acid has been reported to be involved in the iron‐induced synaptic response (Mateos et al., 2012) and it is well known that myoglobin is one of the main iron deposits in mammals, therefore a higher myoglobin content could be related to higher iron releases and, consequently, higher *PLD2* activation.

The third DEG involved in lipid metabolism, *ECH1*, is one of the few down‐regulated in the High MB group. This gene encodes an enzyme that hydrates short‐ and medium‐chain enoyl‐CoA and is related to upregulation of β‐oxidation (Bahnson et al., 2002). Lower expression of this gene was also observed in indigenous Chinese pigs compared to Yorkshire, which also showed better meat quality parameters, such as color, than Yorkshire pigs (Li et al., 2016).

The oxidation–reduction mechanisms are closely related to the generation of the four redox states of myoglobin (DMB, OMB, MetMB, and COMB), which take place mainly within the mitochondria. Antioxidants and secondary reactive products of lipid oxidation named ROS are well known to influence the color stability of pork, affecting the a* parameter, and causing rancidity taste (Faustman et al., 2010; Li et al., 2016; Suman & Poulson, 2013). Moreover, an excessive amount of ROS may possibly damage the mitochondria and cells due to interactions with proteins, lipids, and nucleic acids; also, an increase in the oxidation of mitochondrial fatty acid could increase DNA damage (Cooke et al., 2003). The processes involved on lipid oxidation such as fatty acid elongation and unsaturated fatty acid biosynthesis also affect meat color and rancidity (Li et al., 2016; Wood et al., 2008). The reactive products of lipid oxidation contribute to the discoloration of meat by precipitating MB oxidation, while antioxidants such as vitamin C and E play a protection role and enhance meat color (redness) and lipid stability of lipids (Faustman et al., 2010, Suman & Poulson, 2013). In the same way, *antioxidant action of vitamin C* (Table 4) is one of the most significant pathways in our study, where two of the DEGs upregulated on pigs with higher MB content (*PLA2G10* and *PLD2*) are involved. Expanding on this idea, Li et al. (2016) found that other DEGs involved in the metabolism of lipid oxidation could promote myoglobin oxidation, thereby accentuating cited dark brown color of the meat. Lastly, all this evidence suggests that strategies focused on restrain lipid oxidation can reduce rancidity and improve color stability.

Several genes involved in the innate *(MX2*) and adaptive immune response (*CD209*, *SLA1*, *SLA7*, *LGALS3*, and *LGALS9*) and the inflammatory response (*CHST1*) were also upregulated in the High MB group. While the innate immune system acts as the first line of defense, the adaptive immune system represents the second line, involving T lymphocytes and other cells. The adaptive system provides protection and produces antigen‐specific antibodies from pathogens (Hubler & Kennedy, 2016). *MX dynamin‐like GTPase 2 (MX2*) encodes a protein that has been recognized in the immune defense against virus infection and in the response to interferon α (Goujon et al., 2013; Kane et al., 2013; King et al., 2004). Some studies have described the pivotal antiviral activity also in pigs (Albarracín et al., 2017; Sasaki et al., 2014).

*DC*‐*SIGN (CD209)* encodes a protein with a role in the regulation of T‐cells proliferation (Ryan et al., 2002). In the same way, our functional analyses revealed that *CD209* is associated with the biological process of T‐cell proliferation (GO: 0042098, Table 2). A recent genomic study in Iberian pigs proposed *CD209* as a good candidate gene due to its association with immune defense and modulation during infection by pathogens (Alonso et al., 2020). Another research showed that this gene was upregulated in Duroc pigs with high contents of intramuscular fat and saturated and monounsaturated fatty acids in the gluteus medium muscle (Cardoso et al., 2018). Increased expression of the *CD209* gene has also been observed in skin biopsies from German shepherd dogs with atopic dermatitis and a relationship between this CD209 protein and inflammation has been suggested (Tengvall et al., 2020).

*Src‐like adaptors 1* and *7* (*SLA*‐*1* and *SLA*‐*7*) genes belong to a family that encodes proteins of the porcine major histocompatibility complex. Galectin 3 and galectin 9 (*LGALS3* and *LGALS9*) are S‐type lectins with affinity for β‐galactoside that have antimicrobial activity against bacteria and fungi. In our functional transcriptome study *LGALS3* was involved in the T‐cell proliferation GO_BP_ (Table 2) and is also implicated in the IPA network 1 related to *immunological disease* function (Table 3). Moreover, a porcine transcriptome study recognized *LGALS3* as a relevant innate immune gene expressed in healthy pigs (Snyman et al., 2014). Finally, *CHST1*shows the greatest differences in expression levels (Table 1). This gene encodes a member of the keratin sulfotransferase family of proteins that catalyzes the sulfation of the proteoglycan keratin and generates l‐selectin ligands which are pro‐inflammatory agents (Li et al., 2001).

Two other DEGs upregulated in the High MB group are *EFEMP1* and *COL12A1*. *EFEMP1* encodes a glycoprotein of the extracellular matrix, EGF‐containing fibulin‐like extracellular matrix protein 1. *EFEMP1* is involved in GO_BP_ related to cartilage and connective tissue development. This glycoprotein is part of the fibulin family, which are proteins that modulate cell morphology, growth, adhesion, and motility (Gallagher et al., 2005). Transcriptome and methylome analyses in different pig breeds have suggested that *EFEMP1* is involved in growth and developmental processes (de Yang et al., 2016; Hou et al., 2020; Puig‐Oliveras et al., 2014) and has also been associated with higher human height (Kemper et al., 2012). This gene has also been described as a regulator of hypoxia in Tibetan pigs (Jia et al., 2016). SNP effects of *EFEMP1* on oleic acid have been reported in Wagyu × Angus beef (Zhang et al., 2012); however, no effect of this gene on oleic acid has been observed in pigs. *Collagen type XII alpha 1 chain (COL12A1)* belongs to the collagen family of genes and encodes a protein that plays a key role in organizing the structure of the extracellular matrix and fibrils of collagen. Collagen proteins are the elementary constituent of extracellular matrix. An upregulation of the *COL12A1* gene has been observed when the muscle expression was compared between crossbred Duroc × Iberian with Iberian piglets (Óvilo et al., 2014). Furthermore, Duroc × Iberian crossbreed pigs have less myoglobin content than purebred Iberian pigs (Clemente et al., 2012). Our results indicated an upregulation of the *COL12A1* gene in the High MB group, which is somehow contradictory to the cited studies; however, it is worth to mention that the current study was carried out in Iberian pigs with an average slaughter age of 17 months and in Óvilo et al. (2014), the authors analyzed the transcriptome of piglets with 28 days. Therefore, in pigs with a high MB content there is a greater expression of these two genes involved in the development of connective and cartilaginous tissues.

The upstream analysis predicted a series of regulatory factors that are not necessary differentially expressed in the studied muscle tissue. Several of these regulators (MAPK1, IL1RN, PRL, and SPI1) were identified as master regulators (Table 6). A master regulator is a molecule at the top of a regulatory hierarchy and is expressed at the inception of a developmental lineage, participating in the regulation of multiple downstream genes (Chan & Kyba, 2013). One of the most important factors recognized as a master regulator is mitogen‐activated protein kinase 1 (MAPK1), which has been recognized as a transcriptional regulator involved in the differentiation of porcine myocytes and interrupting the development of adipocytes (Wang et al., 2017), as well as in cellular pathways such as proliferation, differentiation, transcription, cell motility, and apoptosis (Nishida & Gotoh, 1993; Vomastek et al., 2008). This regulator was predicted to be activated in meat samples with lower MB content, regulating the expression of the *FES*, *HBA1*, *LGALS3*, and *MX2* genes, among other.

The master regulator *Spi‐1 proto‐oncogene* (*SPI1*) was activated in the High MB group (Table 6). Figure 5 represents a causal network hypothesis that could explain the regulatory mechanism of SPI1 on four genes *CD209*, *FES*, *LILRB3*, and *VAV1*. *SPI1* would activate the expression of *CD209* that is upregulated in the High MB group, which has previously been linked to with immune defense and inflammation. This protein regulates the expression of multiple genes involved in the immune metabolism (Gangenahalli et al., 2005), moreover, the *SPI1* gene has been related to the regulation of B cells (B lymphocytes), T cells, and myeloid cells (Imoto et al., 2010). Therefore, an upregulation of *SPI1* would promote adhesion of the immune cells’ mechanism. Finally, the *SPI1* gene has been related to the regulation of adipogenesis in porcine transcriptome studies (Li et al., 2011; Wei et al., 2015), thus, the relationship between lipid metabolism and immune system was again observed.

The results shown here indicate that the Iberian pigs with a high myoglobin content show an upregulation of genes codifying pro‐inflammatory proteins. Genes involved in lipid metabolism pathways were also related to the immune system, which reinforce the possibility of a double key function of these DEGs. According to our results, animals with higher myoglobin content seem to have activated lipid oxidation, and we could hypothesize that an increase in oxidation would induce hemoglobin gene expression and augment the myoglobin content in LD muscle, since, as has been suggested, hemoglobin and myoglobin have a protective effect against oxidation. However, further functional studies should be carried out to support this evidence.

## CONCLUSIONS

In this study, 57 DEGs in the transcriptome of LD muscle were identified in Iberian pigs with divergent breeding values for myoglobin content. The functional analyses carried out have revealed that the DEGs codify proteins involved in processes related to inflammation, lipogenesis, and immune defense. These results suggest that pro‐inflammatory proteins, involved in lipid oxidation, could be inducing the expression of the *HBA1* gene, and increasing the myoglobin content since both hemoglobin and myoglobin content have a protective effect against oxidative stress. Polymorphisms located in regulatory regions of the cited DEGs could be associated with their altered expression and, therefore, they could be used in marker‐assisted selection. Finally, the most promising candidate gene underlying the variation in myoglobin content seems to be *HBA1*; therefore, further studies searching for polymorphisms mapped in this gene and association studies with myoglobin content should be carried out.

## CONFLICT OF INTEREST

The authors Luisa Ramírez and Gema Matos are employees of the enterprise “Sánchez Romero Carvajal” and Miguel Ángel Fernández‐Barroso, Yolanda Núñez, Juan María García‐Casco and María Muñoz were employees of the “Instituto Nacional de Investigación y Tecnología Agraria y Alimentaria—INIA” when the study was carried out. Therefore, we declare no conflicts of interest regarding the writing of this manuscript.

## Supporting information

Fig S1

Fig S2

Fig S3

Table S1‐S4

## Data Availability

The data that support the findings of this study are openly available in Gene Expression Omnibus repository at https://www.ncbi.nlm.nih.gov/geo/query/acc.cgi?acc=GSE178915, reference number GSE178915.
