# Supplementary figures and images for: Understanding the role of myoglobin content in Iberian pigs fattened in an extensive system through analysis of the transcriptome profile
Source: Anim Genet. 2022 Mar 30;53(3):352–67. doi: 10.1111/age.13195 (PMC9314091; doi:10.1111/age.13195)

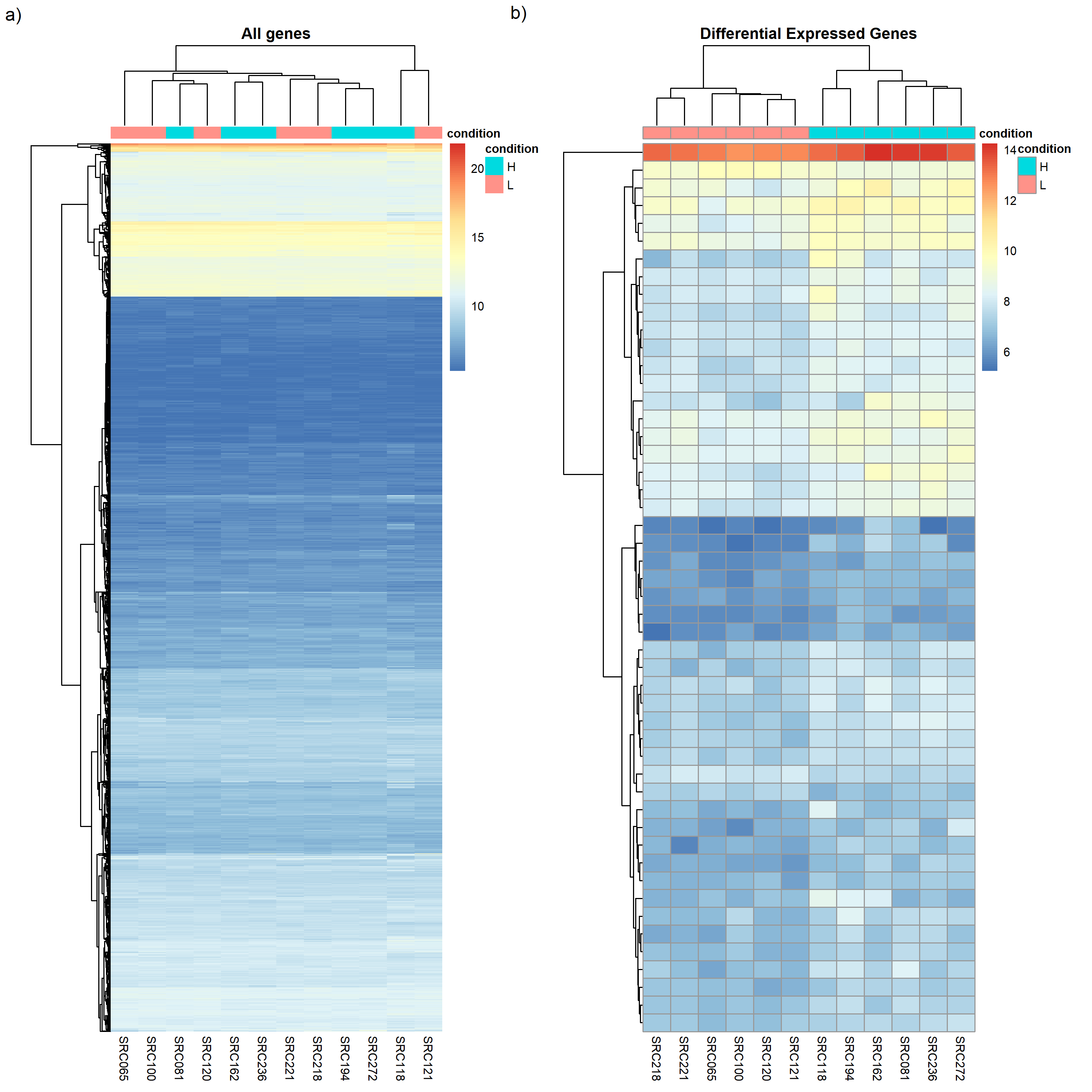

Supplement: Supplementary file 1 — Fig S1 [file AGE-53-352-s003.tiff]

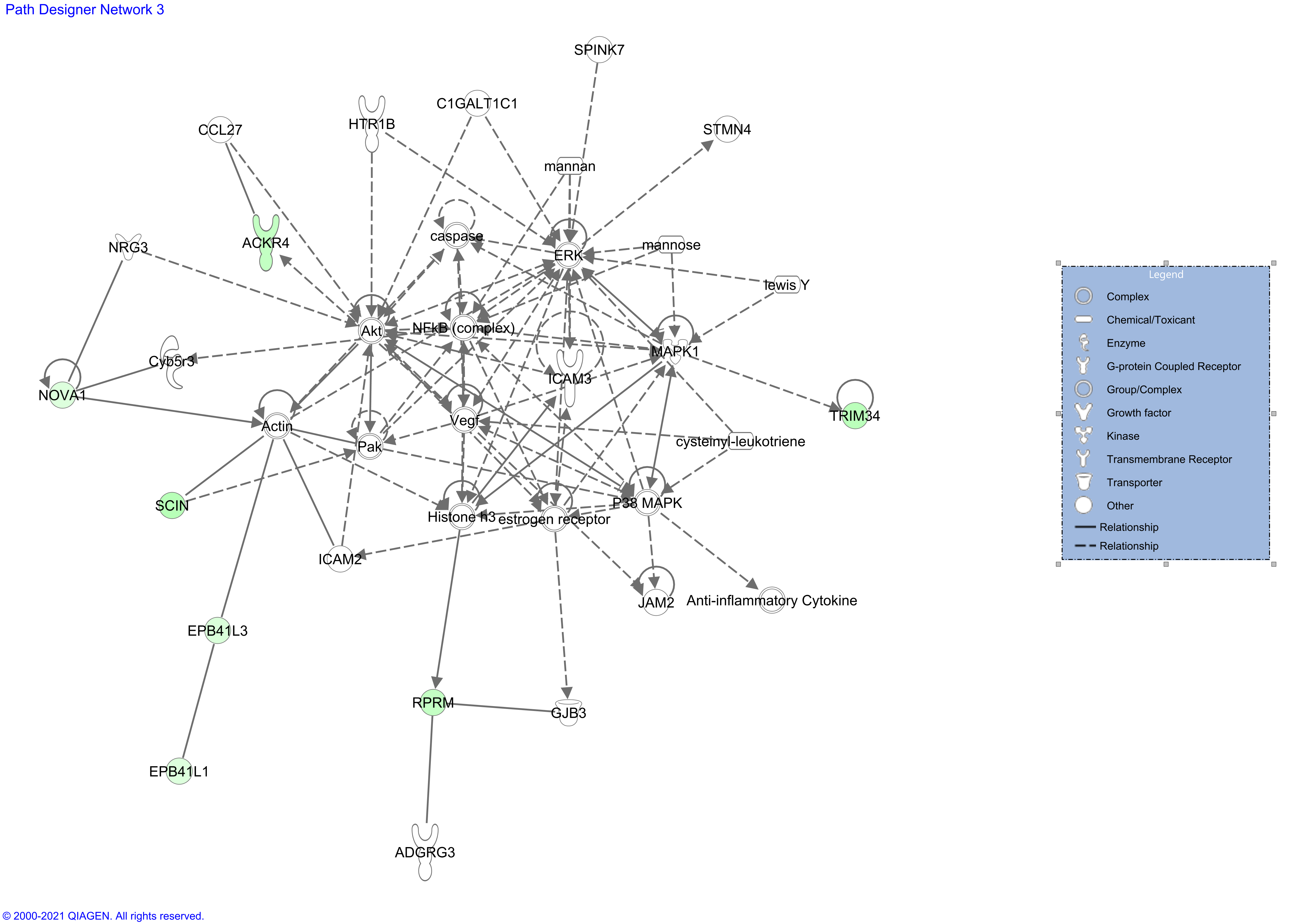

Supplement: Supplementary file 2 — Fig S2 [file AGE-53-352-s001.tiff]

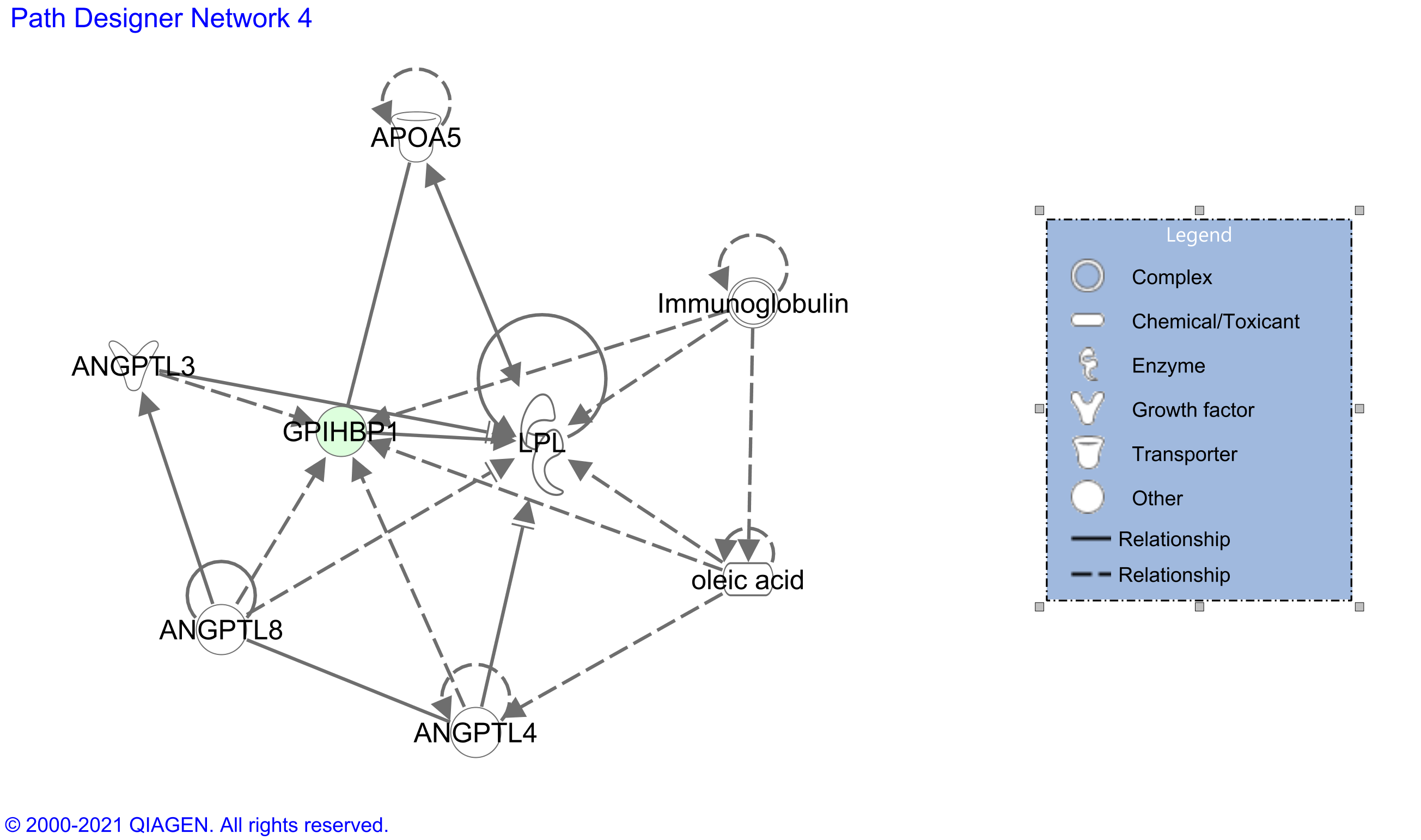

Supplement: Supplementary file 3 — Fig S3 [file AGE-53-352-s004.tiff]
